# Improvement of Superficial and Deep Cutaneous Microcirculation Due to Axillary Plexus Anesthesia Impaired by Smoking

**DOI:** 10.3390/jcm10102114

**Published:** 2021-05-14

**Authors:** Talia Bosselmann, Jonas Kolbenschlag, Ole Goertz, Peter Zahn, Lukas Prantl, Marcus Lehnhardt, Björn Behr, Alexander Sogorski

**Affiliations:** 1 Center of Plastic, Aesthetic, Hand and Reconstructive Surgery, University Hospital of Regensburg, 93053 Regensburg, Germany; lukas.prantl@ukr.de; 2 Department of Hand, Plastic, Reconstructive and Burn Surgery, BG Unfallklinik Tuebingen, Eberhard Karls University Tuebingen, 72076 Tuebingen, Germany; jonaskolbenschlag@googlemail.com; 3 Department of Plastic Surgery and Hand Surgery, Burn Center, BG Universitätsklinikum Bergmannsheil Bochum, Ruhr-University Bochum, 44789 Bochum, Germany; pc.martin-luther@pgdiakonie.de (O.G.); marcus.lehnhardt@bergmannsheil.de (M.L.); bjoern.behr@bergmannsheil.de (B.B.); alexander.sogorski@rub.de (A.S.); 4 Department of Plastic, Reconstructive and Aesthetic Surgery, Hand Surgery, Martin-Luther-Hospital, 14193 Berlin, Germany; 5 Department of Anesthesiology, Intensive Care Medicine, Palliative and Pain Medicine, BG Universitätsklinikum Bergmannsheil Bochum, Ruhr-University Bochum, 44789 Bochum, Germany; Peter.Zahn@bergmannsheil.de

**Keywords:** microcirculation, cutaneous perfusion, axillary plexus anesthesia, regional anesthesia, reconstructive microsurgery, smoking

## Abstract

Background: Understanding microvascular physiology is key to any reconstructive procedure. Current concepts in anesthesia increasingly involve regional peripheral nerve blockade during microvascular reconstructive procedures. Whereas favorable effects on perfusion due to these techniques have been reported earlier, little evidence focusing on its effects in most peripheral vascular compartments is available. Methods: A total of 30 patients who were to receive axillary plexus blockade (APB) were included. Microcirculatory assessment of the dependent extremity was conducted utilizing combined laser-Doppler flowmetry and white light spectroscopy. Two probes (1–2 and 7–8 mm penetration depth) were used to assess changes in microcirculation. Results: APB resulted in significant changes to both superficial and deep cutaneous microcirculation. Changes in blood flow were most prominent in superficial layers with a maximum increase of +617% compared to baseline values. Significantly lower values of +292% were observed in deep measurements. Consecutively, a significant enhancement in tissue oxygen saturation was observed. Further analysis revealed a significant impairment of perfusion characteristics due to reported nicotine consumption (max Bf: +936% vs. +176%). Conclusion: Cutaneous microcirculation is strongly affected by APB, with significant differences regarding microvascular anatomy and vascular physiology. Smoking significantly diminishes the elicited improvements in perfusion. Our findings could influence reconstructive strategies as well as dependent perioperative anesthetic management.

## 1. Introduction

Regional anesthesia, such as axillary plexus anesthesia, has evolved as a standard procedure in the current practice of perioperative analgesia. The safety and efficacy of the procedure has been demonstrated in multiple examinations as wells as in the absence of adverse effects on the conducted surgery [1]. The German Speaking Society for Microsurgery of Peripheral Nerves and Vessels recommends the utilization of regional anesthesia techniques in the context of microsurgical reconstructive procedures according to an expert opinion-based consensus statement [2,3]. Derived from the available literature, it is commonly accepted that regional anesthesia has a beneficial influence on perfusion dynamics as well as on local microcirculation [4,5,6]. However, despite earlier publications already acknowledging the benefits of regional anesthesia in the context of microsurgical interventions, such as digital replantation, there is only limited evidence available from clinical trials with an emphasis on its effects on free flap perfusion and specifically, microcirculatory characteristics [7,8,9].

Most of the conducted studies reported an increase in local perfusion by vasodilation of the greater arteries and accompanying veins as a result of sympatholytic effects. Reduced vascular tone due to sympathetic blockade, which otherwise represents one major vasoconstrictive agent, may also reduce vasospasm of microvessels and transplanted tissues. In previous works, Wenger et al. demonstrated the vasodilatory effects of axillary plexus blockage with enhanced microcirculation regarding blood flow and tissue oxygenation of the finger pulp prior to scheduled hand surgery [6]. Continued postoperative measurements also showed a sustained improvement in microcirculatory parameters for at least 6 h after inception of the anesthesia [5].

A deep understanding of the microvasculature and its perfusion physiology has always been a cornerstone in reconstructive surgery. Nowadays, super-microsurgery and its accompanying new concepts such as perforator-to-perforator anastomosis in free tissue transfer highlight the importance of microcirculation, its disturbance and potential treatment options even more [10,11]. Thus, an understanding of the perfusion physiology of the subdermal plexus and dermal capillary network has gained the attention of reconstructive microsurgeons [12,13].

The aim of our presented study was to further examine the influence of axillary plexus anesthesia via sympathetic blockade on microcirculatory changes in the dependent extremity in humans prior to scheduled hand surgery. In order to represent the complexity of the microvascular network, we focused on the assessment of microcirculation in superficial and deep dermal/subdermal tissue layers with an explorative approach. Our primary hypothesis included the assumption of a different microvascular response with regard to variant vessel anatomy. Beyond that, further analysis of our data could provide an insight into the affectation of microvascular response due to active smoking, which represents a common habit within the European civilization and is known to induce peripheral vascular disease.

## 2. Materials and Methods

The presented study was approved by the local ethics committee of the Ruhr-University Bochum (Reg. number 5176-14, 4 August 2014) and carried out in accordance with the Declaration of Helsinki. All actions were conducted at the BG University Hospital Bergmannsheil Bochum, Germany. Informed consent was obtained from all patients before intervention. All measurements were conducted by the same investigator.

For our prospective study, we included 30 patients (18–50 years of age) scheduled for elective hand surgery at the Department of Plastic Surgery and Hand Surgery, BG University Bergmannsheil Bochum. Exclusion criteria involved infection or injury of the hand, chronic regional pain syndrome of the hand, the use of vasoactive medication, history of vascular disease and pregnancy. Active smoking was defined as daily consumption of cigarettes (>5/daily) for at least one year.

Heartrate (bpm), blood pressure (mmHg) and general oxygen saturation were measured prior to any intervention.

**Figure 1:**
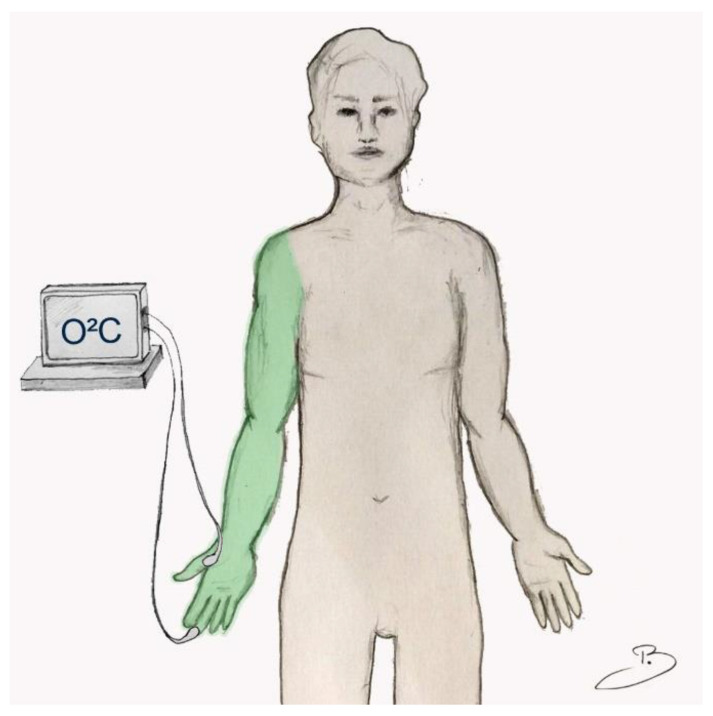
Study se-up: two probes of an “O2C-device” (^©^LEA Medizintechnik, Gießen, Germany) were attached to the palmar skin of the finger pulp (penetration depth 1–2 mm) and the thenar prominence (penetration depth 7–8 mm) for continuous assessment of microcirculatory changes. After a 10 min period of strict rest, the ipsilateral axillary plexus blockade was conducted (marked in green).

Microcirculatory changes as a result of single-shot axillary plexus anesthesia were continuously assessed with the use of an O2C-device (^©^LEA Medizintechnik, Gießen, Germany) [14]. Comprehensive analysis of perfusion was carried out with combined laser Doppler and white light spectroscopy assessments for changes in blood flow (Bf), postcapillary tissue oxygen saturation (StO_2_) and relative hemoglobin content (rHb) [15]. Two probes with different penetration depths were placed and secured on the palm of the ipsilateral hand. A superficial dermal probe (1–2 mm) was attached to the digital pulp of the middle finger, and a deep subdermal probe (7–8 mm) was centered onto the thenar prominence (Figure 1).

Ultrasound-guided single-shot axillary plexus anesthesia was conducted by specialized board-certified anesthesiologists according to a standardized protocol. A 2:1 mixture of 2%-prilocaine and 1%-ropivacaine was used for injection in all cases. Intervention started after a 10-min period of strict rest, with patients positioned in a supine position.

Loss of sensory and motor function (pain, temperature discrimination and movement) due to anesthesia was evaluated by repetitive clinical examinations before and after completion of the procedure. In addition, an inspiratory gasp maneuver was carried out to test for loss of function of the sympathetic nervous system.

### Statistical Analysis

For statistical analysis, Microsoft Excel and IBM SPSS commercially available software were used. All descriptive parameters were presented as mean values ± SD.

Microcirculatory changes as a result of the intervention were calculated as relative changes compared to baseline values (BL). Statistical significance was determined by the confidence interval method. Data are shown as mean and 95 % confidence intervals. For correction of short-term artifacts of continuous microcirculation measurements (one data point per second), these data were separated into corresponding intervals of 5 min length, and linear regression analysis was performed to create a corrected slope. The time of intervention was summarized as one interval, regardless of the elapsed time until completion.

All parameters were checked for normal distribution, and paired students’ *t*-tests were used for comparison of superficial vs. deep measurements and smoking vs. non-smoking, if appropriate. Otherwise, the nonparametric Wilcoxon rank test was chosen.

## 3. Results

Perfusion data of 12 women and 18 men with a mean age of 34.6 years (range: 20–50) were included in the analysis. A mean body mass index of 25.5 kg/m^2^ (±3.6) was calculated. Active smoking was reported in 43.3% of cases. The mean blood pressure of the participants was 130.0 (±14.7)/73.2 (±8.2) mmHg, and global oxygen saturation was 98.3% (±1.5).

The mean volume of infiltrated local anesthetic was 20.63 mL (±2.23) of 2%-prilocaine and 10.63 mL (±1.97) and 1%-ropivacaine. Completion of the brachial plexus anesthesia took 8.35 min (±3.7).

### 3.1. Microcirculatory Measurements

Please see Table 1 for a complete overview of the microcirculatory data.

#### 3.1.1. Blood Flow (Bf)

**Figure 2:**
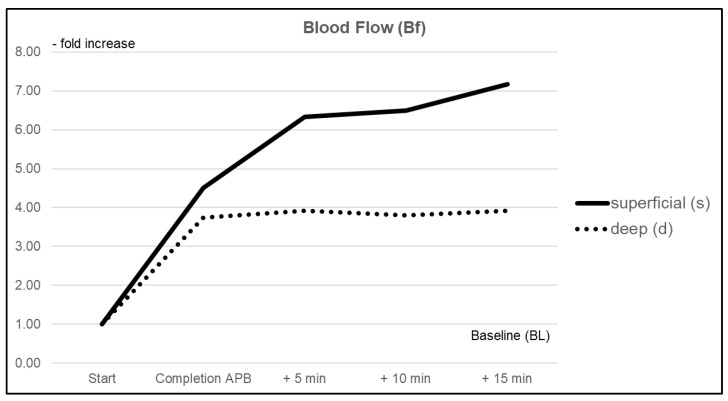
Relative changes in mean blood flow (Bf) within superficial (pulp) and deep (thenar eminence) tissue layers over the duration of the investigation.

Blood flow (Bf) immediately increased due to axillary plexus anesthesia in both the superficial dermal and deep subdermal layers by +351% (CI 2.349–6.676) and +274% (CI 0.568–6.920), respectively. A further increase was observed until the end of the measurements, with a maximum change of +617% (CI 4.122–10.211) in the superficial measurement and +292% (CI 1.346—6.497) for deep measurements during the 15 min follow-up interval. Comparison between both probes showed a significantly more pronounced improvement in superficial vs. deep layers (*p* < 0.05) (Figure 2).

#### 3.1.2. Postcapillary Tissue Oxygen Saturation (StO_2_)

**Figure 3:**
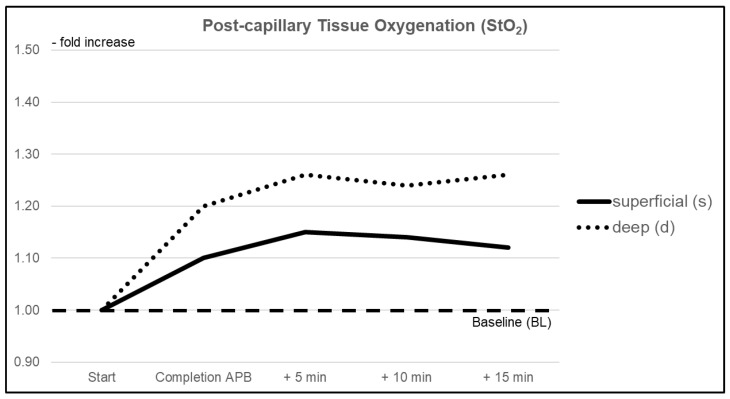
Relative changes in mean post-capillary oxygen saturation (StO_2_) within superficial (pulp) and deep (thenar eminence) tissue layers over the duration of the investigation.

Tissue oxygen saturation was significantly increased within the superficial tissue layers by +10% (CI 1.023–1.167) right after completion of the intervention. A further increase was observed over the following 15 min with a maximum change of +15% (CI 1.049–1.245). Immediate subdermal changes in the StO_2_ values did not reach statistical significance. During the continued observation, a significant maximum increase of +26% (CI 1.054–1.475) compared to BL was reached. There was no significant difference between both measurement sites regarding changes in StO_2_ values due to the intervention at any time (Figure 3).

#### 3.1.3. Relative Hemoglobin Content (rHb)

**Figure 4:**
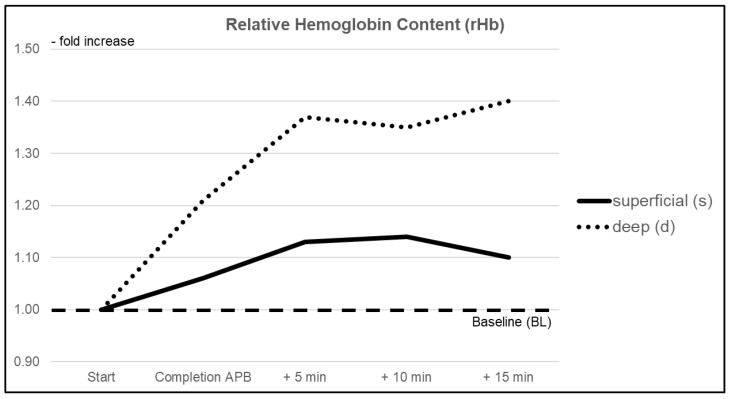
Relative changes in mean relative hemoglobin content (rHb) within superficial (pulp) and deep (thenar eminence) tissue layers over the duration of the investigation.

Relative hemoglobin content (rHb) within superficial layers increased by +6% (CI 0.984–1.138) immediately after completion of the intervention. Changes reached significance during the follow-up period, with a maximum increase of +14% (CI 1.088–1.184). At the subdermal level, an increase of +21% (CI 1.072–1.433) was observed after completion of the intervention, with a further increase of +40% (CI 1.236–1.558) compared to BL at the end of the follow-up interval. Differences between rHb changes reached statistical significance (*p* < 0.05) during the follow-up period at least 10 min after completion of the intervention (Figure 4).

### 3.2. Microcirculatory Analysis: Smoker vs. Nonsmoker 

Please see Table 2 for a complete overview of the microcirculatory data.

**Figure 5:**
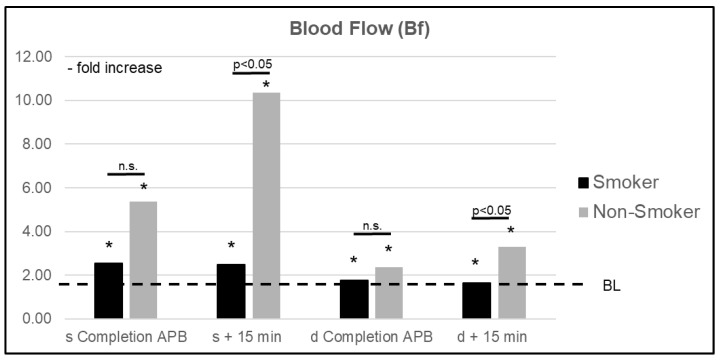
Relative increase in mean superficial (s) and deep (d) blood flow (Bf) in smokers vs. nonsmokers right after completion of the axillary plexus blockade (APB) and after an additional 15 min. BL = baseline value, n.s. = nonsignificant, * significant change compared to BL.

**Figure 6:**
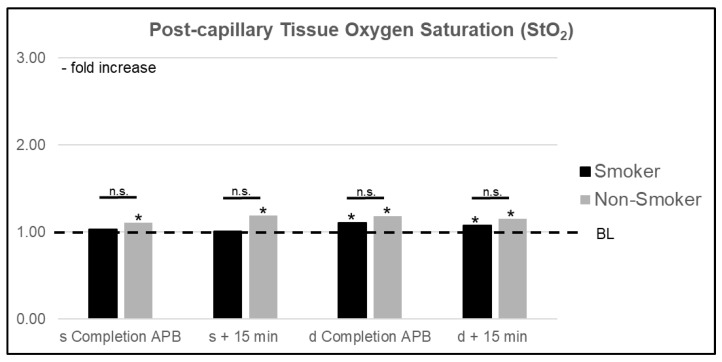
Relative increase of mean superficial (s) and deep (d) post-capillary oxygen saturation (StO_2_) in smokers vs. nonsmokers right after completion of the axillary plexus blockade (APB) and after an additional 15 min. BL = baseline value, n.s. = nonsignificant, * significant change compared to BL.

A comparison of smokers (s) vs. nonsmokers (ns) showed significant differences (*p* < 0.05) in perfusion changes resulting from axial plexus anesthesia. An improvement in superficial Bf was significantly diminished in s, with an increase of +149% (CI 1.667–3.314) vs. +936% (CI 5.597–15.118) in ns. A similar relationship was observed in deep layers with a maximum increase of +77% (CI 1.059–2.474) in s compared to +230% (CI 2.580–4.015) in ns. Whereas superficial StO_2_ values in s were not significantly altered, ns showed significant changes resulting from the anesthesia, with a maximum increase of +21% (CI 1.058–1.364). In deep layers, an improvement of StO_2_ values in s equaled that of ns. In superficial layers, rHb values were significantly elevated in ns by a maximum of +18% (CI 1.118–1.246) compared to BL. The maximum increase in s was 7% (CI 1.010–1.122). Similar to StO_2_ values, changes in rHb reached statistical significance compared to BL in both s and ns for deep measurements, but no significant difference was found between groups (Figure 5 and Figure 6).

## 4. Discussion

Our investigation of changes in cutaneous microcirculation due to regional anesthesia clearly demonstrates the importance of neural signaling in the regulation of perfusion dynamics within the dermal and subdermal compartments. As a result of axillary brachial plexus blockade, a significant increase in cutaneous blood flow and tissue oxygenation as well as relative hemoglobin content was observed. Whereas the improvement in local blood flow was significantly more pronounced within the superficial dermal layer, changes in tissue oxygenation and relative hemoglobin content were most prominent within the subdermal compartment. A comparison between smokers and nonsmokers revealed a significant impairment of microcirculatory improvements resulting from axillary brachial plexus blockade due to active smoking.

Our results are in line with previous work from Wenger et al., who conducted a prospective analysis of the effects of regional anesthesia on cutaneous microcirculation [5,6]. Whereas their investigation focused on the quantification and course of the elicited effects, our goal was to analyze acute effects within different tissue layers and, therefore, different vascular territories.

Cutaneous vascular anatomy includes a rich network consisting of two horizontal plexuses on a superficial sub-papillary level (1–2 mm in depth), which are fed by a deeper (sub-)dermal plexus (7–8 mm in depth) [16]. Regulation of the vessel diameter and therefore cutaneous blood flow, is subjected to a complex interaction of neurohumoral signaling, which, amongst others, involves sympathetic reflex cascades and local endothelial mechanisms [17]. Due to axillary plexus anesthesia, interruption of the sympathetic signaling abrogates its vasoconstrictive effects, which ultimately leads to peripheral vasodilation and an increase in perfusion [18,19].

Based on our findings, the increment in perfusion was more pronounced in the most peripheral superficial part of the vascular network (sub-papillary/papillary plexus), with a consecutive increase in tissue oxygen saturation. A microstructural analysis of the human cutaneous anatomy via electron microscopy revealed characteristic differences in the architecture of the vessels corresponding to their location within the skin. Whereas superficial subpapillary arterioles are usually less than 25 µm in diameter and consist of 1–2 layers of smooth muscle cells (SMC) accompanied by pericytes, the arterioles of the deep horizontal plexus adjacent to the subcutaneous fatty tissue are of much larger caliber (>50 µm), with 5–8 layers of SMC. Although not fully understood, pericytes, which are also present within the deep dermis, are believed to act as vasomotor cells capable of the regulation of vessel diameter [20,21]. Similar to the arterioles, the accompanying veins within the deep (sub-)dermis are of much larger caliber, collecting the blood from a wider range of smaller superficial sub-papillary venules. Corresponding to those anatomical differences, our data show a greater increase in local relative hemoglobin concentration (rHb) within the deeper layers as a result of a generally enhanced state of perfusion. Due to the enhanced Bf, a higher volume of oxygenated blood is directed to the deep venous compartments, so deep StO_2_-values are also increasing. Since the oxygen consumption of dependent cells remains unchanged and total blood volume is higher within the deep layers, changes in StO_2_-values are even more pronounced compared to superficial layers.

Whereas anatomical differences in the vascular network can partially explain our observations, it is likely that there are also functional differences between the superficial and deep plexuses based on the impact of sensory innervation and the autonomic nervous system. In a prior study, Roddie et al. observed a higher oxygen content in blood samples collected from the superficial (primarily skin) compared to deeper (primarily muscle) veins during local heat stress. The increased oxygen content due to an improved perfusion resulting from heat stress was attenuated by a prior nerve blockade proximal to the heating site as well as by sympathectomy [18]. Previous work by Kellogg et al. showed that cutaneous vasoconstriction due to cold stress was abolished after the application of a noradrenergic neuron-blocking agent (bretylium tosylate) [22]. Moreover, functional regulation of skin blood flow via neuronal transmission depends on the body site [18]. Beyond that, there is an active system of cutaneous vasodilation, which has not been fully revealed yet but is believed to consist of complex interactions between (partly unknown) neurotransmitters as well as endothelial mechanisms [17].

Further analysis of our collected data revealed an impairment of the elicited effects in patients who reported active smoking. Previous research demonstrated that chronic as well as acute cigarette smoking significantly affect endothelial function within the human cutaneous microcirculation with consecutively diminished blood flow capacities [23,24]. The marked difference between smokers and nonsmokers that is demonstrated by our presented data can therefore point to the complex mechanistic background of vascular physiology. Whereas axial plexus anesthesia induces vasodilation by suppression of sympathetic vasoconstrictive signaling to smooth muscle cells and pericytes, a staged endothelial-dependent mechanism can nearly abolish the resultant effects. Whether this is caused by an acutely impaired signaling, specifically alterations in metabolic pathways, or results from anatomical variances within capillary morphology due to chronical inflammatory processes is beyond our examination [25]. Additionally, our study lacked a more precise definition of active smoking. It is likely that the disturbance in tissue microcirculation could depend on the daily number of consumed cigarettes as well as differences in the overall duration of active consumption that could impact the severity of vascular disease and perfusion disorders.

Li et al. conducted a dose–response study on the effects of different concentrations of ropivacaine during supraclavicular blockage of the upper extremity blood flow [26]. Briefly, they could demonstrate that there was a dose-dependent effect with increased blood flow changes resulting from higher concentrations of the applied anesthetic agent. Furthermore, the authors concluded that full blockage of the sympathetic vasoconstrictive transmission requires a higher concentration than complete sensory blockade. Based on the results published by Wenger et al., the more pronounced changes in cutaneous blood flow in our current study might be the result of the different composition of the anesthetic agents utilized as well [6].

There is only limited and, in part, conflicting evidence focusing on the effects of regional anesthesia on perfusion, specifically, microcirculation in the field of reconstructive microsurgery. Whereas regional anesthesia appears favorable for reconstructive procedures by means of the aforementioned effects and is generally considered safe, only a few studies were conducted to assess circulatory changes in free tissue transfer due to this anesthetic procedure [27,28]. Moreover, some authors already questioned the advantages of regional anesthesia, for example, in epidural/peridural techniques, based on its resultant changes in general blood flow distribution with consecutive risk of steal-phenomena [29]. Whereas our investigation focused on acute changes due to a single-shot plexus blockade, continuously applied anesthetics via a peripheral nerve catheter might have additional effects on long-term modulation of dependent microcirculation. A continuing enhancement in local microperfusion independent from adverse systemic effects could ultimately lead to improved wound healing, especially in regions of otherwise compromised microcirculation. In summary, further research is required to examine the effects of regional anesthetic procedures on perfusion characteristics of free microsurgical transplants via comprehensive analysis of local microcirculation, thus facilitating specific utilization of these techniques depending on the reconstructive strategy.

## 5. Conclusions

Axillary plexus anesthesia directly affects skin perfusion in dependent tissues in humans. Active smoking leads to a significant impairment of microcirculatory response to regional anesthesia. The amount of observed changes in cutaneous blood flow showed significant differences between the superficial and the deep (sub-)dermal plexuses. Our findings point out the importance of vascular anatomy and the complex mechanistic background of its controlling physiology. Further research is warranted to observe the effects of continuously applied anesthetics on microcirculation within transplanted tissues. With respect to currently evolving concepts in reconstructive microsurgery, especially at the sub-dermal/dermal level, our observations could influence reconstructive strategies as well as dependent perioperative anesthetic management.

## Figures and Tables

**Table 1:** Microcirculatory analysis.

	Relative Change (CI)		*p* (s vs. d)
	Superficial (s)	Deep (d)	
**Blood Flow (Bf)**			
Completion of APB	**4.51 (2.349–6.676)**	3.74 (0.568–6.920)	**0.037**
+5 min	**6.33 (4.079–8.588)**	**3.92 (1.401–6.446)**	**0.002**
+10 min	**6.49 (4.169–8.809)**	**3.80 (1.369–6.229)**	**0.001**
+15 min	**7.17 (4.122–10.211)**	**3.92 (1.346–6.497)**	**0.001**
**Post-capillary Tissue Oxygen Saturation (StO_2_)**			
Completion of APB	**1.10 (1.023–1.167)**	1.20 (0.987–1.406)	0.428
+5 min	**1.15 (1.049–1.245)**	**1.26 (1.051–1.469)**	0.299
+10 min	**1.14 (1.030–1.246)**	**1.24 (1.076–1.399)**	0.102
+15 min	**1.12 (1.008–1.236)**	**1.26 (1.054–1.475)**	0.102
**Relative Hemoglobin Content (rHb)**			
Completion of APB	1.06 (0.984–1.138)	**1.21 (1.072–1.344)**	0.349
+5 min	**1.13 (1.075–1.178)**	**1.37 (1.192–1.553)**	0.060
+10 min	**1.14 (1.088–1.184)**	**1.35 (1.191–1.515)**	**0.023**
+15 min	**1.10 (1.043–1.166)**	**1.40 (1.236–1.558)**	**<0.001**

Complete overview of microcirculatory data. significant changes compared to baseline values are marked bold (APB = axillary plexus blockade, CI = 95%—confidence interval).

**Table 2:** Microcirculatory subgroup analysis smoker vs. nonsmoker.

	Relative Change (CI)					
	Superficial		*p* s vs. ns	Deep		*p* s vs. ns
	Smoker (s)	Non-Smoker (ns)		Smoker (s)	Non-Smoker (ns)	
**Blood Flow (Bf)**						
Completion of APB	**2.54 (1.324–3.750)**	**5.38 (1.929–8.841)**	0.245	**1.77 (1.059–2.474)**	**2.37 (1.597–3.140)**	0.394
+5 min	**2.76 (1.837–3.679)**	**8.69 (5.231–12-149)**	**0.011**	**1.72 (1.181–2.257)**	**3.30 (2.580–4.015)**	**0.005**
+10 min	**2.51 (1.698–3.331)**	**9.17 (5.673–12.659)**	**0.012**	**1.62 (1.073–2.166)**	**3.24 (2.538–3.941)**	**0.004**
+15 min	**2.49 (1.667–3.314)**	**10.36 (5.597–15.118)**	**0.006**	**1.65 (1.080–2.218)**	**3.30 (2.604–6.497)**	**0.003**
**Post-capillary Tissue Oxygen Saturation (StO_2_)**						
Completion of APB	1.03 (0.967–1.088)	**1.11 (1.013–1.213)**	0.347	**1.11 (1.035–1.191)**	**1.08 (1.080–1.159)**	0.556
+5 min	1.04 (0.962–1.115)	**1.21 (1.058–1.364)**	0.195	**1.17 (1.035–1.314)**	**1.15 (1.082–1.211)**	0.394
+10 min	1.01 (0.954–1.073)	**1.21 (1.042–1.388)**	0.088	**1.19 (1.055–1.322)**	**1.14 (1.078–1.207)**	0.777
+15 min	1.01 (0.941–1.072)	**1.19 (1.003–1.372)**	0.303	**1.18 (1.041–1.328)**	**1.15 (1.076–1.217)**	0.777
**Relative Hemoglobin Content (rHb)**						
Completion of APB	0.97 (0.803–1.130)	**1.12 (1.080–1.159)**	0.038	**1.30 (1.050-1.559)**	**1.15 (1.004-1.287)**	0.586
+5 min	1.07 (0.983–1.158)	**1.16 (1.104–1.220)**	0.166	**1.44 (1.125–1.750)**	**1.34 (1.126–1.558)**	0.983
+10 min	**1.07 (1.010–1.122)**	**1.18 (1.118–1.246)**	**0.021**	**1.47 (1.196–1.736)**	**1.29 (1.093–1.487)**	0.444
+15 min	1.05 (0.964–1.134)	**1.14 (1.056–1.222)**	0.140	**1.44 (1.174–1.698)**	**1.39 (1.182–1.592)**	0.913

Complete overview of the microcirculatory data. Significant changes compared to baseline values are marked in bold (APB = axillary plexus blockade, CI = 95%—confidence interval).

## Data Availability

The data presented in this study are available on request from the corresponding author. The data are not publicly available due to privacy issues.
